# 4444 The effect of early life antibiotics on gut microbiome and fecal bile acid concentrations in children

**DOI:** 10.1017/cts.2020.432

**Published:** 2020-07-29

**Authors:** Alain Jesus Benitez, Jeffrey S. Gerber, Ceylan Tanes, Kyle Bittinger, Elliot S. Friedman, Hongzhe Li, Frederic D. Bushman, Gary D. Wu, Babette S. Zemel

**Affiliations:** 1 University of Pennsylvania School of Medicine; 2 Children’s Hospital of Philadelphia; 3 University of Pennsylvania

## Abstract

OBJECTIVES/GOALS: The current proposal seeks to investigate the effect of early life antibiotic use in the development of functional gastrointestinal (GI) disorders. We propose that infants exposed to antibiotics will present with gut microbial dysbiosis, changes in fecal bile acid concentrations and develop more GI symptoms compared to unexposed children. METHODS/STUDY POPULATION: We analyzed fecal samples from 174 subjects at 12 months of age, of whom 52 were exposed to antibiotics in their first year of life. Of these, 33 subjects were sampled again at 24 months of age. DNA from 200mg of frozen stool (−80C) was isolated with the Qiagen DNeasy PowerSoil kit. Shotgun libraries were generated using the NexteraXT kit and sequenced on the Illumina HiSeq 2500 using 2x125 bp chemistry. Sequence data were analyzed using the Sunbeam metagenomics pipeline. The abundance of bacteria was estimated using Kraken version 2.0.8. Fecal bile acids will be quantified by liquid chromatography–mass spectrometry (LC-MS). RESULTS/ANTICIPATED RESULTS: Overall bacterial community composition at 12 or 24 months was not associated with antibiotic exposure (PERMANOVA test, Bray-Curtis distance). An increase in *Enterobacteriaceae*, in particular *Escherichia coli*, is a signature of antibiotic-induced dysbiosis, but also of early infant gut. Children with antibiotic exposure had slightly higher abundance of *Escherichia coli* compared to those with no exposure (p = 0.03). At 24 months, the abundance of *Bacteroides caccae*, a commensal gut species, was decreased for children exposed to antibiotics in the first year of life (fdr = 0.02). We will perform further analysis of bile acid modifying bacteria, fecal bile acid concentrations and correlate to GI symptoms. DISCUSSION/SIGNIFICANCE OF IMPACT: Our findings suggest a significant but nuanced impact of early life antibiotic use on the composition of the gut microbiota. The association of antibiotic exposure with *B. caccae* and *E. coli* warrant further attention in the context of the rapidly developing early-life microbiome. CONFLICT OF INTEREST DESCRIPTION: The authors declare no conflicts of interest relevant to this work.

